# Kinematic Modeling and Solutions for Cable-Driven Parallel Robots Considering Adaptive Pulley Kinematics

**DOI:** 10.3390/s26010039

**Published:** 2025-12-20

**Authors:** Zhonghua Hu, Chaowen Deng, Kai Wang, Jianqing Peng

**Affiliations:** 1 School of Mechanical & Automotive Engineering, Liaocheng University, Liaocheng 252000, China; dcw_lcu_edu@163.com; 2 College of Engineering, China Agricultural University, Beijing 100083, China; wkcau@cau.edu.cn; 3 School of Intelligent Systems Engineering, Shenzhen Campus, Sun Yat-sen University, Shenzhen 518107, China; 4 Guangdong Provincial Key Laboratory of Fire Science and Technology, Guangzhou 510275, China

**Keywords:** cable-driven parallel robot, kinematic modeling, adaptive pulley kinematics, kinematic solution, hybrid Levenberg–Marquardt and Genetic algorithm

## Abstract

Although the use of adaptive pulleys enhances the motion characteristics of cable-driven parallel robots (CDPRs), it significantly increases the complexity of the kinematics model. Conventional methods often fail to account for the influence of adaptive pulley motion on cable length variation, making it difficult to establish a precise kinematics model. To deal with the problem, this study presents a kinematic modeling and solution method for CDPRs, which incorporates adaptive pulley kinematics. First, the structural design of the CDPR driven by eight cables is analyzed. Then, the generalized kinematics model and the improved kinematics model with adaptive pulley considerations are established. Furthermore, a hybrid Levenberg–Marquardt and Genetic algorithm is proposed to achieve the efficient and high-precision solution of kinematics equations by combining the rapid global search and precise local optimization. Finally, the proposed method is validated through straight path simulation and elliptical path simulation. The simulation results indicate that the tracking accuracy of the end-effector is better than the 1 × 10^−7^ level for the proposed method.

## 1. Introduction

The cable-driven parallel robot (CDPR) is a typical parallel mechanism. Unlike traditional rigid-link parallel robots, the end-effector of the CDPR is suspended by a flexible cables, which allows the end-effector to move rapidly in the space [1,2,3]. In addition, CDPRs have many advantages over the rigid-link parallel robots in terms of their high load capacity, large workspace and light overall inertia [4,5]. Therefore, CDPRs are widely used in challenging tasks, such as cargo handling [6], microgravity environment simulation [7], medical rehabilitation [8], 3D printing [9,10], and many other fields. Because of this, the research on CDPR design, modeling and motion planning is gaining attention among scholars.

A CDPR is usually composed of a fixed platform, an end-effector and several cables. Generally, one end of each cable is connected to the actuator on the fixed platform, and the other end is attached to the mobile platform [11]. Based on the number of cables and degree of freedom (DoF), CDPRs can be subdivided into under-constrained CDPRs (UCCDPRs) and fully-constrained CDPRs (FCCDPRs) [2]. If the number of cables is less than that of the end-effector’s DoFs, these robots are UCCDPRs; otherwise, they are FCCDPRs. Mattioni et al. [12] designed an overconstrained planar cable-driven parallel robot to perform quasi-static non-contact operations on the plane. Job et al. [13] established the mathematical model of an UCCDPR with four cables to describe its configuration. Based on this, the range of feasible directions for the UCCDPR end-effector at different positions of the workspace was analyzed. Hwang et al. [14] designed a UCCDPR supported by *n* cables and proposed a novel method to generate the equilibrium configuration trajectory of the UCCDPR. For the FCCDPRs, Gagliardini et al. [15] developed a cable-driven parallel robot for industrial purposes. Moreover, the minimum size of the robot was determined by the base anchor position of each cable. To accomplish pick-and-place objects with heavy weight in large workspaces, Lin et al. [16] designed a multiple-degrees-of-freedom cable-suspended robot with a gripper. Wu et al. [17] proposed a reconfigurable cable-driven parallel robot to avoid collision and improve the flexibility of the robot. Although UCCDPRs and FCCDPRs have been both studied, FCCDPRs are a research hotspot currently due to their higher stiffness and higher dynamic characteristics compared with UCCDPRs. Therefore, this study mainly focuses on the FCCDPRs.

In the structure design of FCCDPRs, one or more pulleys are used for guiding cables and reducing abrasion. Therefore, it is highly necessary to consider the influence of the pulley factor when conducting research into FCCDPRs. Zhang et al. [18] established the kinematics of an FCCDPR, considering pulleys, and they further developed its error model and kinematic calibration method. To significantly increase the size of the wrench-feasible workspace, Martin-Parra et al. [19] designed a novel fully-constrained planar robot with three DoFs and proposed an optimization approach to solve the kineto-static equations of the robot. Reference [20] presented the conceptual design of an FCCDPR with pulleys on its base platform and established the error mapping model considering pulleys. Although the above studies considered the influence of the pulley on the parallel robot, they focused on the error estimation and kinematic calibration of the robot and failed to achieve a kinematic solution directly.

In fact, the kinematics solution issues of FCCDPRs are the inverse kinematics (IK) and the forward kinematics (FK). The IK problem refers to when the pose of the end-effector is given, and then the length of each cable is calculated. In contrast, the FK issue arises when obtaining the pose of the end-effector when the cable lengths are known. For the inverse kinematics of FCCDPRs, there is a direct unique solution, especially without considering the pulley factor [21,22]. Zi et al. [23] discussed the inverse kinematics and statics of a rehabilitation CDPR. Based on this, the optimal design was achieved for the robot’s waist twist and lower limb traction devices. At present, the inverse kinematics approach is always used to analyze the workspace of FCCDPRs [24,25].

Unlike the IK problem, it is challenging to establish the forward kinematics of FCCDPRs, owing to over-determination with respect to the distribution of cables. As the number of cables in the FCCDPRs increases, the challenges become greater. To address this issue, researchers have conducted extensive studies on the forward kinematics of FCCDPRs. Pott and Schmidt [26] formulated forward kinematics as an optimization problem in which the optimal solution was obtained by minimizing the potential energy of cables. To solve the direct kinematics of a CDPR with deformable cables, Merlet [27] developed a generic numerical continuation scheme. In this method, the cable model depended on physical parameters, and it was formulated such that a deformable cable would simplify to a non-deformable one at the parameter limits. For a sagging CDPR, Briot and Merlet [28] converted the problem of solving forward kinematics into a fixed-time Bolza problem. Additionally, they demonstrated that singularities of the forward kinematics model of the CDPRs were the limits of stability. With respect to the same problem in [28], Baskar et al. [29] reformulated the Irvine cable model to solve the global forward kineto-statics of large CDPRs with sagging cables. Here, an iterative strategy was proposed for the root accumulation that employed random monodromy loops. This method tracked circuits instead of lone paths, thereby potentially identifying multiple roots for nonlinear, non-algebraic, and complex-analytic systems. Based on the kinematic solution, the control strategies of CDPRs have attracted more attention among researchers [30,31]. This indicates that the kinematic solution of CDPRs is a core issue.

As discussed above, the existing research commonly ignores the influence of adaptive pulleys during kinematics modeling, despite their significant effect on model accuracy. In addition to this, the mathematical model for the forward kinematics essentially constitutes a system of overdetermined equations, which poses a significant challenge for obtaining efficient solutions. To tackle this issue, the kinematics model of the cable-driven parallel robot was first established, in which the effect of the adaptive pulley on cable length variation has been considered. Furthermore, the kinematic solution method based on the hybrid Levenberg–Marquardt and Genetic algorithm is proposed for the cable-driven parallel robot. Finally, the straight trajectory and elliptic trajectory are adopted as the desired paths of the end-effector for verifying the correctness and effectiveness of the proposed method.

The remainder of this study is organized as follows. Section 2 analyzes the overall structure and design principle of the cable-driven parallel robot. In Section 3, the generalized kinematics model and the improved kinematics model with adaptive pulley considerations are both established, and the forward or inverse kinematics equations were obtained. Based on the hybrid Levenberg–Marquardt and Genetic algorithm, Section 4 proposes an effective kinematic solution method. In Section 5, the proposed method is verified through the simulation of a straight path and an elliptical path. Based on the analysis in the previous sections, the last section summarizes the study.

## 2. Design of the Parallel Robot

### 2.1. Overall Structural Design

**Figure 1:**
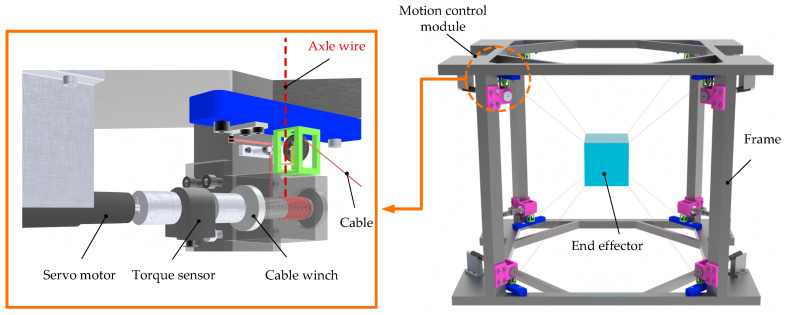
The overall structure of the cable-driven parallel robot.

**Figure 2:**
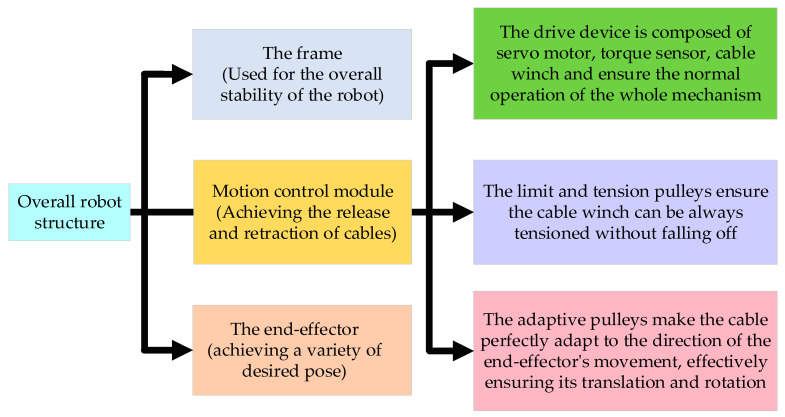
The design scheme of the CDPR.

The mechanical structure of the cable-driven parallel robot (CDPR) is depicted in Figure 1. The robot comprises a frame, an end-effector, and eight motion control modules. Each module has the same structure and consists of a servo motor, torque sensor, cable winch reel, limit and tension pulley, and adaptive pulley. These modules are symmetrically arranged at the eight vertices of the frame. The servo motor serves as the power source for the motion control module. The motor’s output torque is transmitted to the cable winch reel and drive it to rotate. The torque sensor continuously monitors the actuator’s output torque and sends real-time data to the control unit, ensuring the control accuracy of cables. Each cable tip is fixed to the end-effector, enabling its translation and rotation through the coordinated release and retraction of the cables. Due to the symmetrical structural design of the robot, its space utilization, control accuracy, stiffness, and load capacity are significantly enhanced. The adaptive pulley is a core component of the motion control module, capable of rotating around the central axis of its base in response to the movement of the end-effector. This rotational ability enables the cable to move synchronously with the end-effector, ensuring its translation and rotation around in three-dimensional space. The structural design principles and the component function of the CDPR are given in Figure 2.

### 2.2. Operating Principle of Motion Control Module

**Figure 3:**
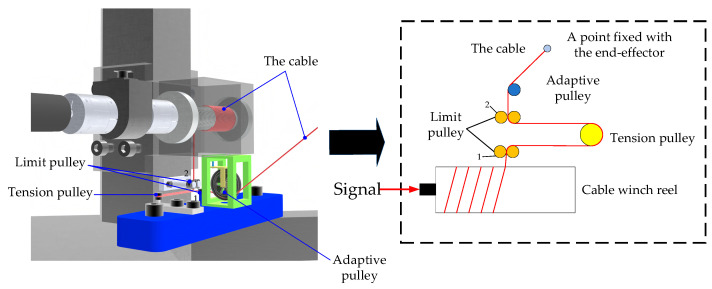
The schematic of the cable path layout.

As shown in Figure 3, the working principle of the cable-driven parallel robot is as follows. The signal from the upper computer is received by servo motors. Then, the servo motor starts and the cable winch reel connected to the servo motor rotates in synchronization with it. The release and retraction of the cable are achieved through the rotation of the cable winch reel. Initially, the direction of the cable is determined by the limit pulley 1, after which the tension pulley enables the cable to maintain tension.

Simultaneously, the limit pulley 2 further guarantees the cable’s orientation. For precisely controlling the cable’s direction, an adaptive pulley is equipped behind the limit pulley 2. After passing through the adaptive pulley, the cable is connected to the end-effector of the CDPR. As a result, the desired motion of the end-effector can be achieved by the pulling and releasing cables.

## 3. Kinematics Modeling of the Robot

The kinematics of the cable-driven parallel robot consists of two parts: the inverse solution of the kinematics and the forward solution of the kinematics. Inverse kinematics means that the position and attitude of the end-effector are known, and then the cable length corresponding to the above pose can be calculated. Forward kinematics involves calculating the position and attitude of the end-effector based on each articulated point position of the robot and its corresponding cable length. In the kinematics analysis of the CDPR, the characteristics of the adaptive pulley model are considered for improving the accuracy of kinematics modeling. In this section, the generalized kinematics model and the improved kinematics model with adaptive pulley considerations are studied. Before the modeling analysis, some assumptions are given as follows.

(a) The cable is handled as a straight line.
(b) The effect of gravity on the cable is neglected.
(c) The cable is in a tensioned state at all times.

### 3.1. Generalized Kinematics Model

**Figure 4:**
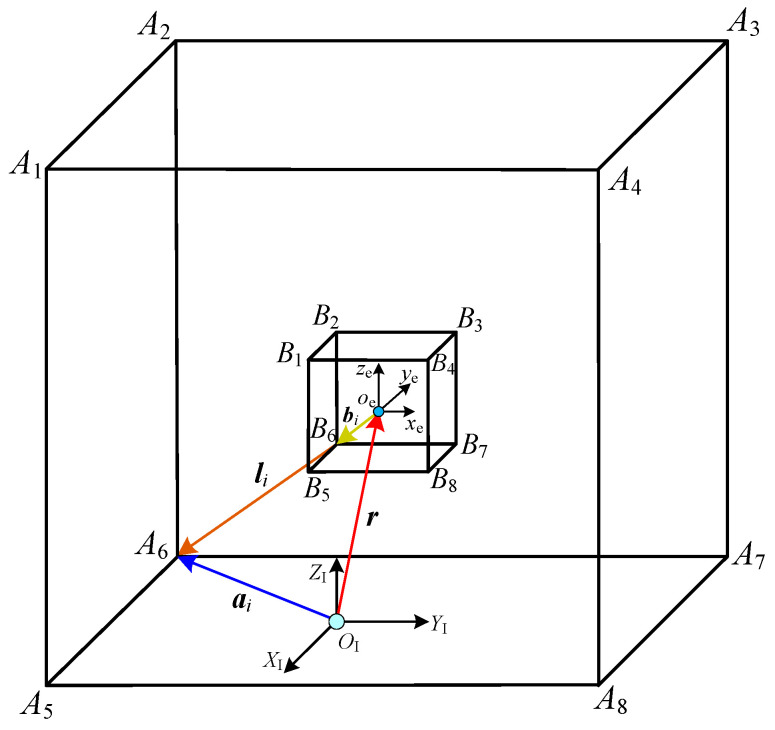
The general model of the cable-driven parallel robot.

The simplified structure model of the cable-driven parallel robot is shown in Figure 4, which mainly consists of a fixed frame, the end-effector and eight cables. The cube *A*_1_~*A*_8_ represents the fixed frame of the CDPR, and each vertex *A_i_* (*i* = 1, 2, …, 8) is the point of cable exit. The other cube *B*_1_~*B*_8_ is the end-effector of the CDPR and its vertices *B_i_* (*i* = 1, 2, …, 8) are connected with the other end of cables (i.e., the *i*th cable connects *A_i_* and *B_i_*). The inertial system {*X*_I_, *Y*_I_, *Z*_I_} is denoted as Σ_I_, which is fixed on the bottom plane center of the fixed frame, and its origin is *O*_I_. {*x*_e_, *y*_e_, *z*_e_} (named by Σ_e_) is the moving coordinate system, which is fixed in the geometric center of the end-effector. The position and attitude of Σ_e_ changes with the movement of the end-effector.

As given in Figure 4, based on the closed vector principle, the *i*th cable vector ***l****_i_* is obtained as follows.

(1) $$l_{i}=a_{i}-r-Rb_{i}$$

where ***a****_i_* is the position vector of point *A_i_* in the inertial system. ***b****_i_* is the position vector of point *B_i_* which is connected with the other end of the *i*th cable and ***b****_i_* is expressed in Σ_e_. ***r*** is the position vector from the origin of Σ_I_ to the origin of Σ_e_. ***R*** is the attitude transformation matrix from Σ_e_ to Σ_I_.

The end-effector’s pose (position and attitude) is defined as *Q* = (*X*_e_, *Y*_e_, *Z*_e_, $\psi_{1}$, $\psi_{2}$, $\psi_{3}$). *X*_e_, *Y*_e_, and *Z*_e_ are the position coordinates in Σ_I_. $\psi_{1}$, $\psi_{2}$, and $\psi_{3}$ represent attitude angles (X–Y–Z Euler angles). Hence, ***r*** and ***R*** can be calculated as follows.

(2) $$\left\{\begin{array}{l} r=\left[\begin{array}{ccc} X_{e} & Y_{e} & Z_{e} \end{array}\right]\\ R=R_{xyz}(\psi_{1},\psi_{2},\psi_{3})=\left[\begin{array}{ccc} c_{\psi_{2}}c_{\psi_{3}} & -c_{\psi_{2}}s_{\psi_{3}} & s_{\psi_{2}}\\ s_{\psi_{1}}s_{\psi_{2}}c_{\psi_{3}}+c_{\psi_{1}}s_{\psi_{3}} & c_{\psi_{1}}c_{\psi_{3}}-s_{\psi_{1}}s_{\psi_{2}}s_{\psi_{3}} & -s_{\psi_{1}}c_{\psi_{2}}\\ s_{\psi_{1}}s_{\psi_{3}}-c_{\psi_{1}}s_{\psi_{2}}c_{\psi_{3}} & c_{\psi_{1}}s_{\psi_{2}}s_{\psi_{3}}+s_{\psi_{1}}c_{\psi_{3}} & c_{\psi_{1}}c_{\psi_{2}} \end{array}\right] \end{array}\right.$$

where $s_{\psi_{i}}=\sin(\psi_{i}),c_{\psi_{i}}=\cos(\psi_{i})$, *i* = 1, 2, 3.

According to Equation (2), the length of the *i*th cable is calculated as follows.

(3) $$l_{i}=\left\|l_{i}\right\|=\sqrt{{\left(a_{i}-r-Rb_{i}\right)}^{Τ}\left(a_{i}-r-Rb_{i}\right)}$$

Setting $l=[l_{1},l_{2},l_{3},l_{4},l_{5},l_{6},l_{7},l_{8}]$, the mapping relationship between the cable lengths and the end-effector’s pose can be expressed as:

(4) $$\left\{\begin{array}{l} \left[r,\;R\right]=FK^{GK}\left(l,a_{i},b_{i}\right),\;\;\mathrm{Forward}\;\mathrm{Kinematics}\\ l=IK^{GK}\left(r,R,a_{i},b_{i}\right),\;\;\;\;\;\;\;\mathrm{Inverse}\;\mathrm{Kinematics} \end{array}\right.$$

where $FK^{GK}(•)$ and $IK^{GK}(•)$ are implicit functions of the forward kinematics and the inverse kinematics for the generalized kinematics model, respectively.

### 3.2. Kinematics Modeling Considering the Adaptive Pulley

Since the eight winch reel modules (four upper modules and four lower modules) have the same mechanical structure, an upper winch reel module and the corresponding lower winch reel module are taken as an example to establish the improved kinematics model of the cable-driven parallel robot.

**Figure 5:**
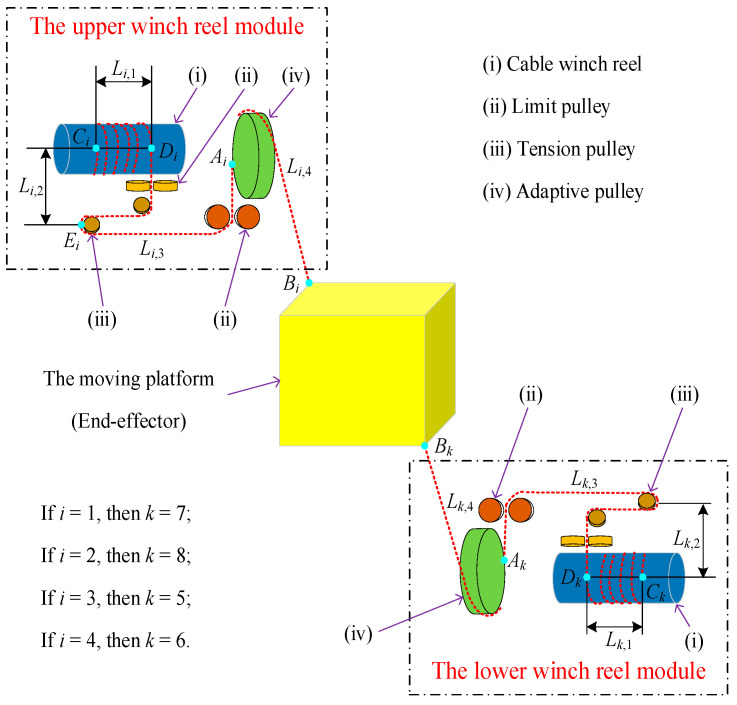
The upper and lower winch modules.

As shown in Figure 5, the cable in the upper winch reel module can be divided into four sections through limit pulley, tension pulley and adaptive pulley. The cable length of the first section is set as *L_i_*_,1_, which is from the cable fixed point *C_i_* to the cable exit point *D_i_* on the winch reel. *L_i_*_,2_ is the cable length of the second segment, that is, from point *D_i_* to the tangency point *E_i_* of the tension pulley. The third cable section is from point *E_i_* to the tangency point *A_i_* of the adaptive pulley and its length is denoted by *L_i_*_,3_. The length of the last cable section is set as *L_i_*_,4,_ that is, from point *A_i_* to the connection point *B_i_* on the end-effector. For the lower winch reel module, the definitions of symbols are the same as in the upper module, except that the subscript *i* is replaced by the symbol *k*. It should be noted that *L_i_*_,2_ and *L_i_*_,3_ are constant, after the structural design of the robot is completed. The values of *L_i_*_,1_ and *L_i_*_,4_ will change with the movement of the end-effector. However, the primary focus of this study is the corresponding relationship between the end-effector pose and *L_i_*_,4_. Even though *L_i_*_,1_ is a variable, it does not affect the subsequent kinematics modeling and solution results. As a result, *L_i_*_,4_ is served as the main research object.

**Figure 6:**
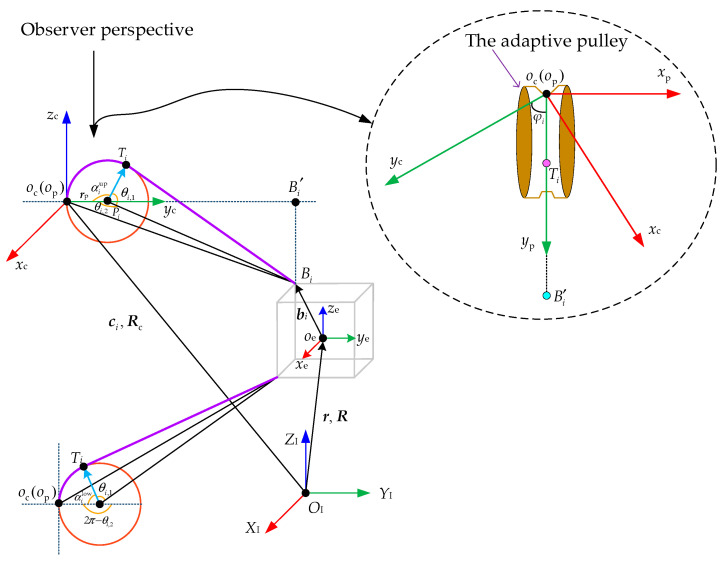
Kinematics model with adaptive pulley.

In order to describe the kinematics model including adaptive pulleys, two coordinate systems {*x*_c_, *y*_c_, *z*_c_} and {*x*_p_, *y*_p_, *z*_p_} are built, as shown in Figure 6. Their origins (*o*_c_ and *o*_p_) are both set at point *A_i_*. In the initial state, the *x*_c_-axis, *y*_c_-axis and *z*_c_-axis are parallel to three axes of {*x*_p_, *y*_p_, *z*_p_}, respectively. The coordinate system {*x*_p_, *y*_p_, *z*_p_} rotates with the movement of the adaptive pulley.

The rotation angle of the adaptive pulley is denoted by $\varphi_{i}$. $T_{i}$ is the cable exit point and $\vec{T_{i}B_{i}}$ is the tangent line of the adaptive pulley. $\alpha_{i}$ is the central angle corresponding to the arc $\overset{⏜}{o_{c}T_{i}}$ on the adaptive pulley. In other words, $\alpha_{i}$ is the wrap angle of the *i*th cable around the adaptive pulley. The value range of $\alpha_{i}$ is divided into two categories due to the different installation locations of adaptive pulleys. One is $\alpha_{i}^{\mathrm{up}}$ that corresponds to the adaptive pulley in upper winch modules and $\alpha_{i}^{\mathrm{up}}\in (90^{\circ },\;\;180^{\circ })$. The other one is $\alpha_{i}^{\mathrm{low}}$ which is associated with the adaptive pulley in lower winch modules and $\alpha_{i}^{\mathrm{low}}\in (0^{\circ },\;\;90^{\circ })$. The point $P_{i}$ is the center of the *i*th adaptive pulley. $\theta_{i,1}$ is the angle between the vector $\vec{P_{i}T_{i}}$ and the vector $\vec{P_{i}B_{i}}$. $\theta_{i,2}$ is the angle between $\vec{P_{i}B_{i}}$ and the vector $\vec{P_{i}o_{c}}$.

When considering the motion of adaptive pulleys, the actual length of the *i*th cable is made up of the lengths of arc $\overset{⏜}{o_{c}T_{i}}$ and line $\vec{T_{i}B_{i}}$. If the lengths of $\overset{⏜}{o_{c}T_{i}}$ and $\vec{T_{i}B_{i}}$ are denoted by $L_{i}^{\mathrm{arc}}$ and $L_{i}^{\mathrm{line}}$, they can be calculated as follows:

(5) $$\left\{\begin{array}{l} L_{i}^{\mathrm{arc}}=\alpha_{i}\left\|r_{p}\right\|\\ L_{i}^{\mathrm{line}}=\left\|\left(r+Rb_{i}\right)-\left(c_{i}+R_{c}R_{z}\left(\varphi_{i}\right)r_{p}-R_{c}R_{z}\left(\varphi_{i}\right)R_{x}\left(-\alpha_{i}\right)r_{p}\right)\right\| \end{array}\right.$$

where ***r***_p_ is the vector form *o*_c_ to *P_i_* at the initial state. Thus, ***r***_p_ = [0, *d*, 0]^T^, the radius vector of the adaptive pulley. Its magnitude is denoted by *d*. ***c****_i_* is the position vector in Σ_I_ and it is a known quantity. ***R***_c_ is the rotation matrix from Σ_c_ to Σ_I_ and ***R****_z_*(*φ_i_*) and ***R****_x_*(*α_i_*) are rotation matrixes about the *z*-axis and *x*-axis, and they are obtained as follows:

(6) $$R_{z}\left(\varphi_{i}\right)=\left[\begin{array}{ccc} \cos\varphi_{i} & -\sin\varphi_{i} & 0\\ \sin\varphi_{i} & \cos\varphi_{i} & 0\\ 0 & 0 & 1 \end{array}\right],\;R_{x}\left(-\alpha_{i}\right)=\left[\begin{array}{ccc} 1 & 0 & 0\\ 0 & \cos\alpha_{i} & \sin\alpha_{i}\\ 0 & -\sin\alpha_{i} & \cos\alpha_{i} \end{array}\right]$$

Unlike the generalized kinematics model that can directly compute the cable lengths if the pose of the end-effector is known, the improved kinematics model requires calculating the angle *φ_i_* and the angle *α_i_* to determine cable lengths. The improved kinematic model refers to the inverse kinematics considering adaptive pulleys movement. The detailed calculation process of the above two angles is given as follows.

When the pose of end-effector and the attitude transformation relationship between Σ_c_ and Σ_I_ is known, the position vector of *B_i_* in the Σ_c_ is determined by:

(7) ocBi→c=RcΤ⋅r+Rbi−ci

Because the frame {*x*_p_, *y*_p_, *z*_p_} moves along with the adaptive pulley and the cable $\vec{T_{i}B_{i}}$ is tangent to the adaptive pulley, $\vec{T_{i}B_{i}}$ is always on the plane *y*_p_*o*_p_*z*_p_. As a result, the projection of the point *B_i_* on the plane *x*_p_*o*_p_*y*_p_ must be on the *y*_p_-axis and it is denoted by ${B_{i}}^{\prime }$ (see Figure 6). The position vector of ${B_{i}}^{\prime }$ in the Σ_c_ is calculated as follows:

(8) ocBi′→c=100010000⋅ocBi→c=100010000RcΤ⋅r+Rbi−ci

According to the definition of dot product, the angle *φ_i_* can be obtained as follows:

(9) φi=arccosocBi′→c⋅010ΤocBi′→c

Furthermore, it is the process of calculating the angle *α_i_*. The vector $\vec{P_{i}B_{i}}$ is obtained as follows:

(10) $$\vec{P_{i}B_{i}}=r+Rb_{i}-c_{i}+R_{c}R_{z}\left(\varphi_{i}\right)r_{p}$$

Based on the previous discussion, $\vec{T_{i}B_{i}}$ is the tangent line of the adaptive pulley. Therefore, the vector $\vec{P_{i}T_{i}}$ is perpendicular to $\vec{T_{i}B_{i}}$ and $\Delta P_{i}T_{i}B_{i}$ is a right triangle. $\theta_{i,1}$ can be obtained by:

(11) $$\theta_{i,1}=\arccos\left(\frac{\left\|\vec{P_{i}T_{i}}\right\|}{\left\|\vec{T_{i}B_{i}}\right\|}\right)=\arccos\left(\frac{d}{\left\|\vec{T_{i}B_{i}}\right\|}\right)$$

According to the cosine theorem of a triangle, $\theta_{i,2}$ is calculated as follows:

(12) θi,2=arccosd2+PiBi→2−ocBi→c22dPiBi→

On basis of the relationship among angles $\alpha_{i}$, $\theta_{i,1}$ and $\theta_{i,2}$, $\alpha_{i}$ can be calculated. However, it should be noted that the angle $\alpha_{i}$ has two different results, i.e., $\alpha_{i}^{\mathrm{up}}$ and $\alpha_{i}^{\mathrm{low}}$, which are obtained as follows:

(13) $$\alpha_{i}=\left\{\begin{array}{l} \alpha_{i}^{\mathrm{up}}=2\pi -\theta_{i,1}-\theta_{i,2},\;\mathrm{Corresponding}\;\mathrm{to}\;\mathrm{upper}\;\mathrm{adaptive}\;\mathrm{pulley}\\ \alpha_{i}^{\mathrm{low}}=\theta_{i,2}-\theta_{i,1},\;\;\;\mathrm{Corresponding}\;\mathrm{to}\;\mathrm{lower}\;\mathrm{adaptive}\;\mathrm{pulley} \end{array}\right.$$

Combining Equations (5), (9) and (13), the length of the *i*th cable is determined as follows:

(14) $$l_{i}=L_{i}^{\mathrm{arc}}+L_{i}^{\mathrm{line}}=d\alpha_{i}+\left\|\left(r+Rb_{i}\right)-\left(c_{i}+R_{c}R_{z}\left(\varphi_{i}\right)\left[\begin{array}{c} 0\\ d\\ 0 \end{array}\right]-R_{c}R_{z}\left(\varphi_{i}\right)R_{x}\left(-\alpha_{i}\right)\left[\begin{array}{c} 0\\ d\\ 0 \end{array}\right]\right)\right\|$$

Here, $\varphi_{i}$ and $\alpha_{i}$ can be obtained based on Equations (9) and (13). Similarly, the pose of the end-effector will be obtained when each cable’s length is known. The following two assumptions are given:

(15) $$\varphi ={\left[\varphi_{1},\varphi_{2},\varphi_{3},\varphi_{4},\varphi_{5},\varphi_{6},\varphi_{7},\varphi_{8}\right]}^{Τ}$$

(16) $$\alpha ={\left[\alpha_{1},\alpha_{2},\alpha_{3},\alpha_{4},\alpha_{5},\alpha_{6},\alpha_{7},\alpha_{8}\right]}^{Τ}$$

The models of forward kinematics and inverse kinematics, which both consider adaptive pulleys, can be established as follows:

(17) $$\left\{\begin{array}{l} \left[\varphi ,\alpha ,r,R\right]=FK^{MK}\left(l,b_{i},c_{i},R_{c}\right),\;\;\;\mathrm{Forward}\;\mathrm{Kinematics}\\ \left[\varphi ,\alpha ,l\right]=IK^{MK}\left(r,R,b_{i},c_{i},R_{c}\right),\;\;\;\mathrm{Inverse}\;\mathrm{Kinematics} \end{array}\right.$$

where $FK^{MK}(•)$ and $IK^{MK}(•)$ are the forward kinematics and inverse kinematics equations for the improved modeling method, respectively.

## 4. Kinematics Solution Based on a Hybrid Levenberg–Marquardt and Genetic Algorithm

### 4.1. The Methodology of the Fusion Algorithm

The cable-driven parallel robot has 6-DOFs, redundantly actuated by eight cables. Due to the existence of redundant constraints, it poses significant challenge for the kinematic solution of the robot, especially solving the forward kinematics.

Although scholars have conducted some research on this issue, some numerical algorithms, such as the Newton–Raphson method and neural network method, have their own limitations in both respects. One is the ability to achieve a kinematic solution on the workspace boundary. The other is the solving accuracy of training samples. In order to address these issues, a hybrid algorithm based on a hybrid Levenberg–Marquardt and Genetic method is proposed to achieve the efficient and high-precision solution of kinematics equations by combining rapid global search and precise local optimization. In the algorithm, the Genetic method is adopted to converge to near the optimal solution rapidly. Based on this, the above results can provide theoretical support for trajectory planning and motion control strategies for cable-driven parallel robots.

According to Equation (17), the specific relationship between the length of each cable, the pose of the end-effector and the angles of adaptive pulleys can be obtained. Therefore, the objective equation of the forward kinematics solution can be established as follows:

(18) $$\left\{\begin{array}{l} \min\left\|G\left(\varphi ,\alpha ,r,R\right)\right\|,\;G\left(\varphi ,\alpha ,r,R\right)=f\left(\varphi ,\alpha ,r,R\right)-l_{d}\\ s.t.\left\{\begin{array}{l} \left({\left\|c_{i}+R_{c}R_{z}\left(\varphi_{i}\right)r_{p}-\left(r+Rb_{i}\right)\right\|}^{2}-{\left\|r_{p}\right\|}^{2}\right)-{\left(l_{d}\left(i\right)-\alpha (i)\left\|r_{p}\right\|\right)}^{2}=0\\ -\frac{\pi }{2}\leq \varphi (i)\leq \frac{\pi }{2}\\ \frac{\pi }{2}\leq \alpha (i)\leq \pi ,\;i=1,2,3,4\\ 0\leq \alpha (i)\leq \frac{\pi }{2},\;i=5,6,7,8 \end{array}\right. \end{array}\right.$$

where $l_{d}$ is the desired length of all cables and it is a known quantity. $f\left(\varphi ,\alpha ,r,R\right)$ represents the function for calculating the length of cables, and it can be obtained using Equation (14). In the same way, the function of the inverse kinematics solution for the robot can also be built by utilizing the approach in Equation (18). Based on the hybrid Levenberg–Marquardt and Genetic algorithm (HLMGA), the process of the forward kinematics solution is listed in Algorithm 1.
**Algorithm 1.** The process for solving forward kinematics based on HLMGA1**Require**$:\;\mathrm{Desired}\;\mathrm{cable}\;\mathrm{lengths}\;l_{d}$2$\mathrm{Initialize}\;\mathrm{the}\;\mathrm{pose}\;(r_{0}$, $R_{0}$$)\;\mathrm{and}\;\mathrm{angles}\;(\varphi_{0}$, $\alpha_{0}$)3**for** *t* = 0 **to** *T* **do**4 Genetic algorithm main loop5  Set the size of population *N*_pop_ and threshold *δ*_1_6  Calculate each individual *fitness*, based on Equation (20) 7  Sort population by *fitness* and select top 50% individuals8  Perform single-point crossover with probability *P*_rc_10  Apply small perturbations with probability *P*_rm_11  Combine selected individuals and mutated individuals12 Levenberg–Marquardt local optimization13  Set scaling coefficient *μ*, amplification coefficient *v* and threshold *δ*_2_14  **for** *k* = 1 **to** *N_L_*_-*M*_ **do**15  $\mathrm{Computing}\;\mathrm{the}\;\mathrm{Jacobian}\;\mathrm{matrix}\;J_{k}$$\mathrm{and}\;\mathrm{the}\;\mathrm{function}\;\mathrm{value}\;G_{k}$
16  Update step ***d****_k_* using Equation (22)17  $\mathrm{Recalculate}\;{\hat{J}}_{k}$
$\mathrm{and}\;{\hat{G}}_{k}$
18  Evaluate step size effectiveness λ, based on Equation (24)19  **if** λ > 0, reduce the value and adjust the amplification factor20  **else if** increase *μ* and increase *v*21  **end if**22  **if**
$\left\|d_{k}\right\|<\delta_{2}$, then, **break**23  **else**$\;\mathrm{if},\;\mathrm{reset}\;X_{e},\;Y_{e},\;Z_{e},\;\psi_{1},\;\psi_{2},\;\psi_{2},\;\varphi_{i},\;\alpha_{i}$, and go to step 1324  **end if**25 **end for**26end for27Output the optimal result

To facilitate further explanation of the proposed method, the detailed process of the forward kinematics solution is given as follows.

### 4.2. Parameter Initialization

(a) Set the size of the fixed frame as that described in Section 2. Set the simulation time (*T*) and the simulation time step (d*T*). Initialize the pose of end-effector and the angles of adaptive pulleys as follows:

(19) $$\left\{\begin{array}{l} r_{0}={\left[\begin{array}{ccc} X_{e}(0) & Y_{e}(0) & Z_{e}(0) \end{array}\right]}^{Τ},\;R_{0}=R_{x}(\psi_{1}(0))R_{y}(\psi_{2}(0))R_{z}(\psi_{3}(0))\\ \varphi_{0}={\left[\begin{array}{cccccccc} \varphi_{1}(0) & \varphi_{2}(0) & \varphi_{3}(0) & \varphi_{4}(0) & \varphi_{5}(0) & \varphi_{6}(0) & \varphi_{7}(0) & \varphi_{8}(0) \end{array}\right]}^{Τ}\\ \alpha_{0}={\left[\begin{array}{cccccccc} \alpha_{1}(0) & \alpha_{2}(0) & \alpha_{3}(0) & \alpha_{4}(0) & \alpha_{5}(0) & \alpha_{6}(0) & \alpha_{7}(0) & \alpha_{8}(0) \end{array}\right]}^{Τ} \end{array}\right.$$

where *X*_e_(0), *Y*_e_(0) and *Z*_e_(0) are the position of the end-effector at the *T* = 0. Its initial attitude angles (X–Y–Z Euler angles) are *X*_e_(0), *Y*_e_(0) and *Z*_e_(0), respectively. α(*i*) is the initial angle of the adaptive pulley.

(b) Set the parameters of the Genetic algorithm. The population size is set as *N*_pop_ = 300. The maximum number of iterations *Maxitr*_heredity_, the crossover probability *P*_rc_ and the mutation probability *P*_rm_ are 50, 0.8, and 0.05, respectively.

(c) Initialize the parameters of the Levenberg–Marquardt algorithm. The initial scaling coefficient *μ* and amplification coefficient *v* are set as *μ* = 0.01 and *v* = 5. The amplification factor *v* is used to increase the value of *μ* when the step size of the Levenberg–Marquardt method is not appropriate. The maximum number of iterations is *Maxitr*_L-M_ = 20. The convergence termination coefficients for the Genetic method and Levenberg–Marquardt are *δ*_1_ and *δ*_2,_ respectively.

### 4.3. Genetic Algorithm Main Loop

(a) Coding and population setup

Through the decimal encoding strategy, the population is randomly generated in the solution space for each time step. Each individual in the population represents a possible variable that includes the pose of the end-effector and the angles of adaptive pulleys. The initial population is generated by adding random noise to ensure diversity and even distribution.

(b) Fitness function calculation

For each individual in the population, its fitness value calculation is the key to solving the forward kinematics rapidly. First, the objective function value is calculated according to the value of each individual, and then the cable length error is obtained. If the above error exceeds the set threshold, a larger penalty term will be introduced and it is denoted by *penalty*, which can let the individual still retain a certain choice possibility but place it at a disadvantage in the choice. Otherwise, the optimized value is obtained. Here, the nonlinear equations representing the solution of the forward kinematics of the cable-driven parallel robot can be obtained using Equation (18). In other words, the pose and angles satisfying $\left\|G\left(\varphi ,\alpha ,r,R\right)\right\|=0$ are the optimal solution of the forward kinematics. However, in the actual solution process, it is difficult to obtain a solution that completely satisfies the above equation, so it can only be sorted according to the sum of fitness values, and the smallest individual is selected to inherit to the next generation. The function values of each connection point are added to obtain the fitness value of the individual, i.e.:

(20) $$fitness=\sum_{i=1}^{8}{\left(G(i)\right)}^{2}$$

where G(i) is the *i*th element of function value G.

(c) Select an operation

The absolute fitness value of each individual is sorted, and the half of the individuals with the least fitness are selected as the parents of the next generation.

(d) Cross operation

For each pair of adjacent parent individuals from 1 to (*N*_pop/_2) − 1, the crossing operation is performed with the probability *P*_rc_.

(e) Mutation operation

For each parent individual from *N*_pop/_2 to *N*_pop_, the mutation operation is performed with the probability *P*_rm_. The variation point is selected randomly, and minimal random disturbance is added to the pose and angles parameters of the point to ensure the diversity of the population and to avoid falling into the local optimal.

(f) Population renewal

For the new individuals, their crossover and mutation are merged with the parent population to form a new population and to continue the next iteration.

### 4.4. Local Optimization by L-M Algorithm

(a) Local optimization after Genetic algorithm

After each iteration of the Genetic algorithm, the optimal individual with the best fitness value is used as the initial value of the Levenberg-Marquardt algorithm.

(b) Jacobian matrix and function values calculation

The Jacobian matrix and the function value can be calculated based on Equation (18). The Jacobian matrix $J_{k}$ reflects the sensitivity of cable lengths with respect to the pose of end-effector and angles of adaptive pulleys. The central difference method can also be used for numerical calculation. Hence, it is derived as follows:

(21) $$J_{k}=\left[\begin{array}{c} \frac{\partial G(1)}{\partial X_{e}},\;\frac{\partial G(1)}{\partial Y_{e}},\;\frac{\partial G(1)}{\partial Z_{e}},\;\frac{\partial G(1)}{\partial \psi_{1}},\;\frac{\partial G(1)}{\partial \psi_{2}},\;\frac{\partial G(1)}{\partial \psi_{3}},\;\frac{\partial G(1)}{\partial \varphi_{1}},\ldots ,\frac{\partial G(1)}{\partial \varphi_{8}},\;\frac{\partial G(1)}{\partial \alpha_{1}}\ldots \frac{\partial G(1)}{\partial \alpha_{8}}\\ \frac{\partial G(2)}{\partial X_{e}},\frac{\partial G(2)}{\partial Y_{e}},\frac{\partial G(2)}{\partial Z_{e}},\frac{\partial G(2)}{\partial \psi_{1}},\frac{\partial G(2)}{\partial \psi_{2}},\frac{\partial G(2)}{\partial \psi_{3}},\frac{\partial G(2)}{\partial \varphi_{1}},\ldots ,\frac{\partial G(2)}{\partial \varphi_{8}},\frac{\partial G(2)}{\partial \alpha_{1}}\ldots \frac{\partial G(2)}{\partial \alpha_{8}}\\ \vdots \\ \frac{\partial G(8)}{\partial X_{e}},\frac{\partial G(8)}{\partial Y_{e}},\frac{\partial G(8)}{\partial Z_{e}},\frac{\partial G(8)}{\partial \psi_{1}},\frac{\partial G(8)}{\partial \psi_{2}},\frac{\partial G(8)}{\partial \psi_{3}},\frac{\partial G(8)}{\partial \varphi_{1}},\ldots ,\frac{\partial G(8)}{\partial \varphi_{8}},\frac{\partial G(8)}{\partial \alpha_{1}}\ldots \frac{\partial G(8)}{\partial \alpha_{8}} \end{array}\right]$$

In addition, the function value G can be obtained using Equation (18) directly. G(i) is the *i*th element of G.

(c) The precise solution calculation

In the iteration process of the *L*–*M* method, the search step size is calculated first, and the parameters including the pose and angles are updated. Then, the new attitude matrix, Jacobian matrix and function values are obtained. Finally, we determine whether the step size meets the termination condition, and if so, stop the iteration. Otherwise, the scale coefficient is adjusted according to the effect of the step size update. The Jacobian matrix and the function value are updated to continue the next iteration. Here, the step size is calculated as follows:

(22) $$d_{k}=-{\left(J_{k}^{Τ}J_{k}+\mu I\right)}^{-1}J_{k}^{Τ}G_{k}$$

where ***I*** is the 12th-order identity matrix. The function value $G_{k}$ represents the deviation between the current cable length and the desired cable length in the *k*th step calculation.

If the step size fails to meet the termination condition, the values of pose and angles should be updated by the following equation:

(23) $$\left\{\begin{array}{l} X_{e}(k+1)=X_{e}(k)+d_{k}(1),Y_{e}(k+1)=Y_{e}(k)+d_{k}(2),Z_{P}(k+1)=Z_{P}(k+1)+d_{k}(3)\\ \psi_{1}(k+1)=\psi_{1}(k)+d_{k}(4),\psi_{2}(k+1)=\psi_{2}(k)+d_{k}(5),\psi_{3}(k+1)=\psi_{3}(k)+d_{k}(6)\\ \varphi_{i}(k+1)=\varphi_{i}(k)+d_{k}(i+6);\alpha_{i}(k+1)=\alpha_{i}(k)+d_{k}(i+14),i=1,2,\ldots ,8 \end{array}\right.$$

Substituting Equation (23) into Equations (18) and (21), the updated Jacobian matrix and the function value can be recalculated.

If the step size $\left\|d_{k}\right\|<\delta_{2}$, the algorithm converges and the iteration is terminated. If not, we calculate the value of the adaptive damped shadow *λ* as follows:

(24) $$\lambda =\frac{G_{k}^{Τ}G_{k}-{\hat{G}}_{k}^{Τ}{\hat{G}}_{k}}{\mu {d_{k}}^{Τ}d_{k}-{d_{k}}^{Τ}J_{k}^{Τ}G_{k}}$$

where ${\hat{G}}_{k}$ is the deviation of cable length in the next moment.

Adjust the proportional coefficient *μ* according to the value *λ* obtained by Equation (24). If *λ* > 0, reduce the value *μ* and adjust the amplification factor *v*. Otherwise, increase the *μ* value and increase the value *v* until $\left\|d_{k}\right\|<\delta_{2}$ is satisfied. Finally, the optimal result is obtained by utilizing the hybrid Levenberg–Marquardt and Genetic algorithm.

## 5. Simulation Study

To validate the proposed method, the simulation model of the cable-driven parallel robot is established by Matlab2020 software. The straight and elliptical paths are used as the desired end-effector trajectories for verifying the forward and inverse kinematic solution. In the simulation, the parameters of the robot are listed in Table 1.

### 5.1. The Simulation Results for Inverse Kinematics

#### 5.1.1. The Straight Path Simulation

**Figure 7:**
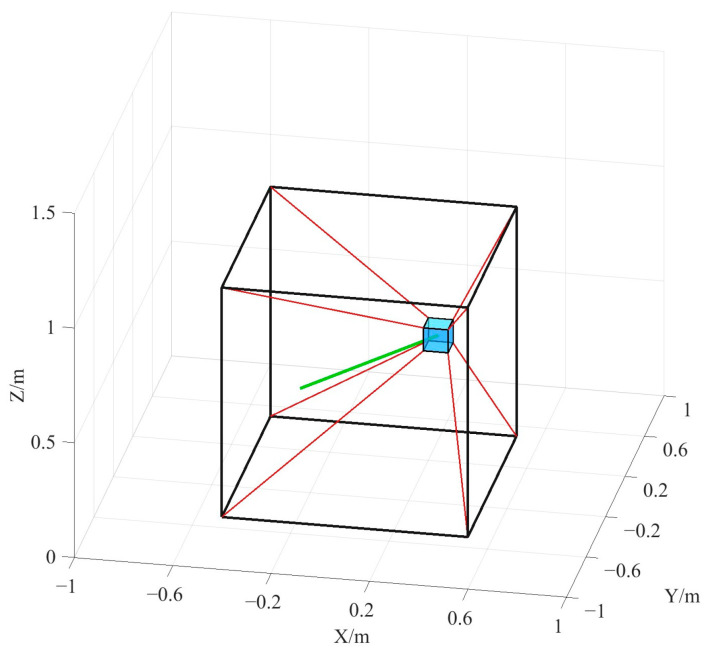
Spatial linear trajectory.

First, the space straight trajectory is taken as the desired path of the end-effector for the simulation, as shown in Figure 7. The total simulation time is *T* = 12 s. The start point and the end point of the straight trajectory are set as follows:

(25) $$\left\{\begin{array}{l} P_{\mathrm{ls}}={\left[\begin{array}{ccc} -0.35\; & 0.35\; & 0.2 \end{array}\right]}^{Τ}m\\ P_{\mathrm{lf}}={\left[\begin{array}{ccc} 0.35\; & -0.35\; & 0.8 \end{array}\right]}^{Τ}m \end{array}\right.$$

Meanwhile, we assume that the end-effector moves at constant speed. Therefore, the real-time position of the end-effector is obtained as follows:

(26) $$P_{l}\left(t\right)=P_{\mathrm{ls}}+\frac{t}{T}\left(P_{\mathrm{lf}}-P_{\mathrm{ls}}\right),\;t\in \left[0,12\right],T=12$$

The desired attitude angles (X–Y–Z Euler angles) of the end-effector are set as [0°, 0°, 0°], which means the end-effector maintains its orientation aligned with the inertial frame.

**Figure 8:**
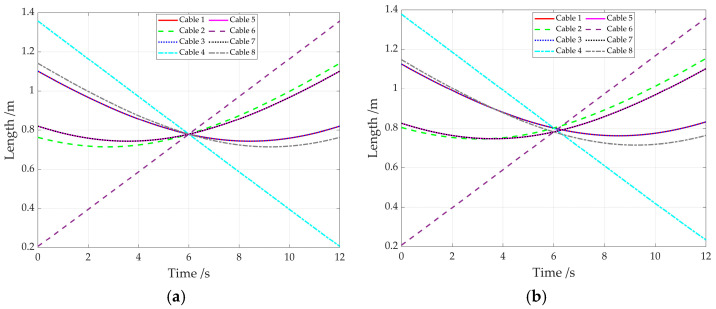
The variations in cable lengths. (**a**) For the generalized kinematics model; (**b**) for the improved kinematics model.

Based on Equations (4) and (17), the lengths of eight cables utilizing the generalized kinematics model or the improved kinematics model that accounts for the adaptive pulley are shown in Figure 8. It can be seen that the variations in cable lengths from both methods exhibit a largely consistent trend, with certain deviations in the specific values.

**Figure 9:**
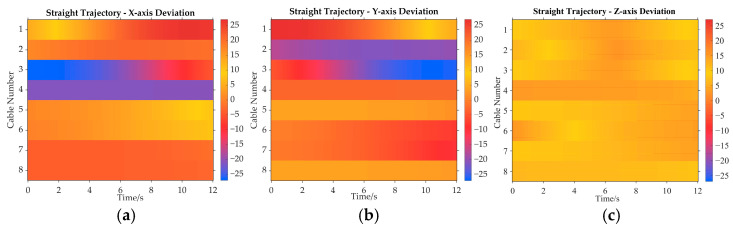
The position deviations of the cable release points for both methods. (**a**) Along the *X*_I_ axis; (**b**) along the *Y*_I_ axis; (**c**) along the *Z*_I_ axis.

The position deviations of the cable release points for both methods are shown in Figure 9. Figure 9a is the position deviation heatmap along the *X*_I_ axis of the inertial system. Cable 1 and Cable 2 exhibit positive deviations, and Cable 3 and Cable 4 display negative deviations. The position deviation heatmaps along the *Y*_I_ axis and *Z*_I_ axis are Figure 9b and Figure 9c, respectively. All cables are towards positive deviations, as shown in Figure 9c.

Additionally, it can be concluded that the actual cable departure point on the pulley is affected continuously during the movement of adaptive pulleys, and the deviation is roughly 0.02 m. The comparison of cable release point position deviations presented in Figure 9 aims to demonstrate that the traditional method, by neglecting the geometric variations introduced by the motion of the adaptive pulley, leads to a significant deviation (approximately 0.02 m) between the calculated cable release point positions and the actual cable release point positions. This deviation is not random noise but a systematic error introduced by model simplification. If the traditional method is used for the actual control directly, a non-negligible error between the theoretical trajectory and the actual trajectory of the end-effector would arise, severely limiting the robot’s performance in high-precision tasks such as precise trajectory tracking.

**Figure 10:**
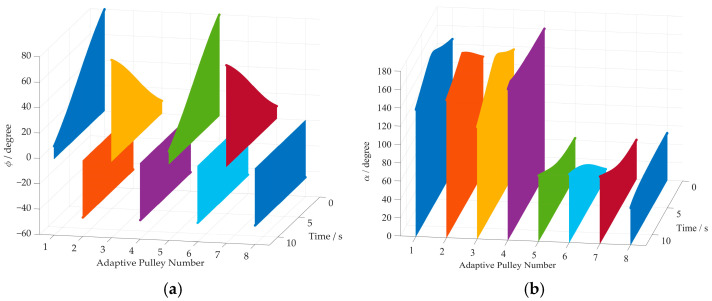
The rotation angles and the wrap angles of cables in straight path simulation. (**a**) The rotation angle of each adaptive pulley; (**b**) the wrap angles of cables around adaptive pulleys.

The 3D surface diagram for the rotation angles and wrap angles of cables is shown in Figure 10. From Figure 10a, one can see that the variations in rotation angles for all adaptive pulleys are bounded between −60° and 80° at each time step of the simulation. Moreover, the wrap angle of each cable around the corresponding adaptive pulley is shown in Figure 10b. It can be observed that the wrap angles of Cables 1–4 range from 90° to 180°. They fall within the range of 0° to 90° for Cables 5–8. The correctness of the improved kinematics model is further validated by these results.

#### 5.1.2. The Ellipse Path Simulation

Here, an elliptic trajectory is adopted to verify the inverse kinematics. The position of the ellipse’s center is set as $O_{\mathrm{ell}}=[0.1,\;0.15,\;0.6]$. The normal vector of the plane which the ellipse is on is $n_{\mathrm{ell}}=[0,\;0,\;1]$. The major semi-axis of the ellipse is along the *X*_I_ axis and its length is 0.3 m. The length of the minor semi-axis is 0.25 m and it is along the *Y*_I_ axis. Therefore, the desired elliptic trajectory at every moment is obtained as follows.

(27) $$P_{\mathrm{ell}}\left(t\right)={\left[\begin{array}{ccc} P_{\mathrm{ell}\_x} & P_{\mathrm{ell}\_y} & P_{\mathrm{ell}\_z} \end{array}\right]}^{Τ}$$

where:

(28) $$\left\{\begin{array}{l} P_{\mathrm{ell}\_x}=0.1+0.3\cos\left(2\pi t/T\right)\\ P_{\mathrm{ell}\_y}=0.15+0.25\sin\left(2\pi t/T\right)\\ P_{\mathrm{ell}\_z}=0.6 \end{array}\right.\;t\in \left[0,12\right],T=12$$

**Figure 11:**
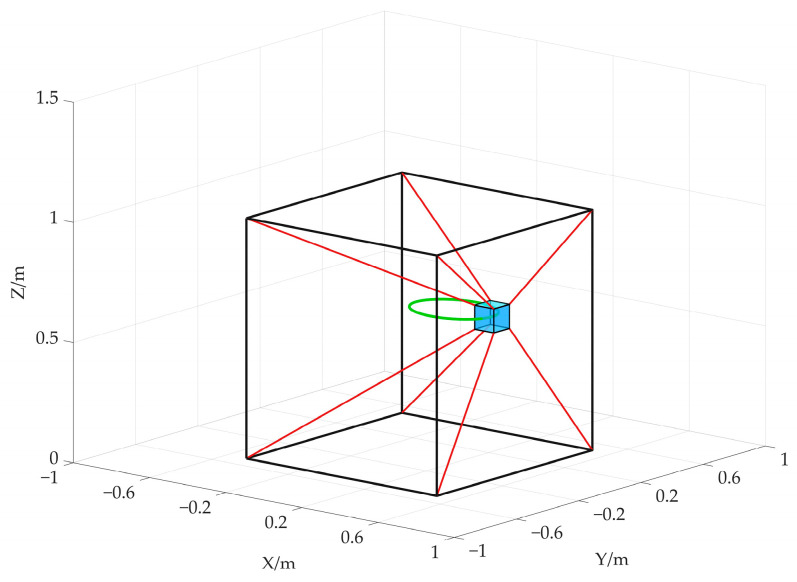
Spatial elliptic trajectory.

In addition, the desired attitude angles (X–Y–Z Euler angles) of the end-effector are also set as [0°, 0°, 0°]. The space ellipse trajectory is as shown in Figure 11.

**Figure 12:**
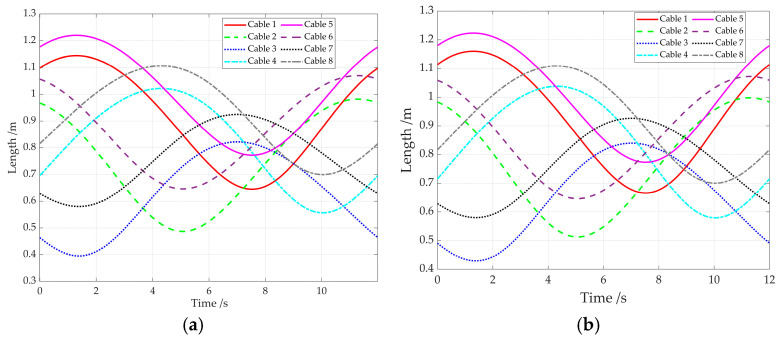
Cable length variations. (**a**) For the generalized kinematics model; (**b**) for the improved kinematics model.

For the elliptic path simulation, the cable length variations are given in Figure 12. They exhibit clear periodic characteristics for the generalized kinematics model and the improved kinematics model. This is because the end-effector moves along the elliptical trajectory, enabling its pose to change periodically along the major and minor axes of the ellipse.

**Figure 13:**
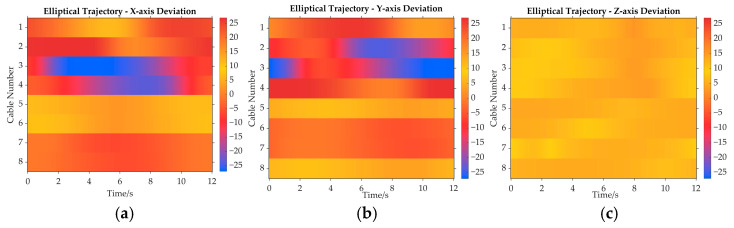
Cable release position deviations in the ellipse path simulation. (**a**) Along the *X*_I_ axis; (**b**) along the *Y*_I_ axis; (**c**) along the *Z*_I_ axis.

As shown in Figure 13, through the heatmap analysis, the position deviation of each cable’s departure point is visually characterized and compared. Here, Figure 13a shows the comparison of position deviations along the *X*_I_ axis for the traditional method and the improved method, considering the adaptive pulley effect. Figure 13b,c represents the position deviations along the *Y*_I_ axis and *Z*_I_ axis, respectively. Similarly to the straight path simulation, the position deviations are all approximately 0.02 m in the ellipse path simulation. As a result, these results demonstrate that, if the traditional method is employed in practical computations, it fails to calculate the cable length variations of the actual model. This finding further validates the correctness of the improved method.

**Figure 14:**
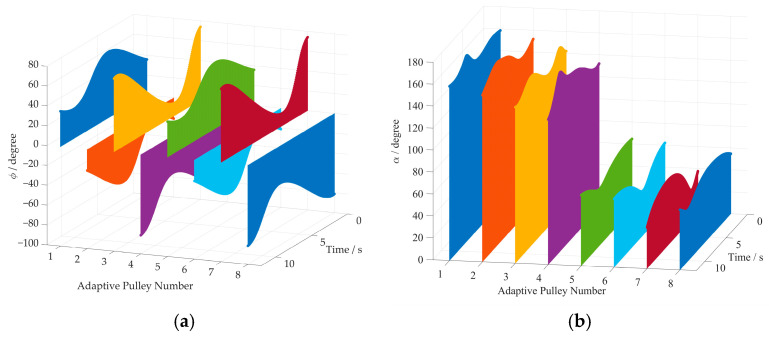
The rotation angles and the wrap angles of cables. (**a**) The rotation angle of each adaptive pulley; (**b**) the wrap angles of cables around the adaptive pulleys.

The rotation angles of all adaptive pulleys are shown in Figure 14a. Figure 14b shows the variation in the cable wrap angle around each adaptive pulley. Like the linear trajectory simulation, the values of the rotation angles and cable wrap angles of the adaptive pulleys are bounded within their defined intervals, which verifies the effectiveness of the proposed method.

### 5.2. Simulation Results for Forward Kinematics

For further verification, the simulation model of the cable-driven parallel robot is also established. The model parameters are the same as those used in the inverse kinematics simulation. In the forward kinematics simulation, the cable lengths are taken as known inputs to calculate the position and attitude of the end-effector. Here, the cable length data are generated by the straight and elliptical trajectories used in Section 5.1. In this section, we first use HLMGA to solve the forward kinematics of the straight-line trajectory and the elliptical trajectory, and then we use the particle swarm optimization algorithm to solve the forward kinematics of the straight-line trajectory.

#### 5.2.1. Forward Kinematics Simulation Based on the Straight Path via the HLMGA Algorithm

**Figure 15:**
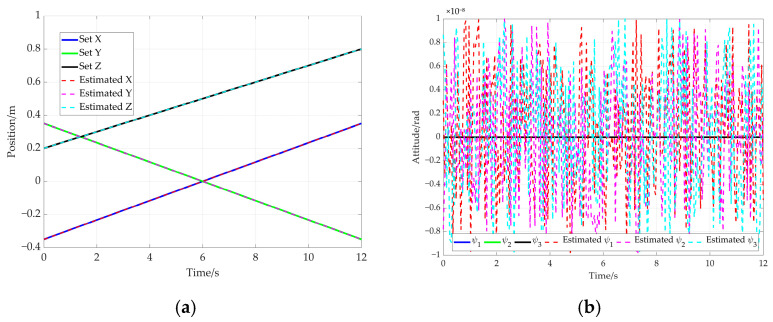
Comparison between preset values and solved values for the end-effector pose. (**a**) End-effector position comparison; (**b**) end-effector attitude comparison.

**Figure 16:**
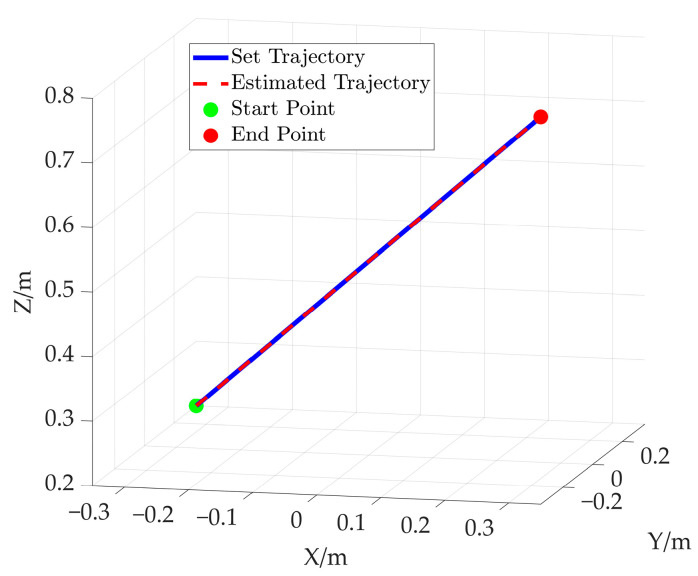
Comparison between the preset straight trajectory and estimated straight trajectory.

Based on the hybrid Levenberg–Marquardt and Genetic algorithm, the pose of the end-effector is obtained based on the cable length data. The comparison between the preset value of end-effector’s pose and the solved value produced using the proposed method is shown in Figure 15. In this figure, the solid line represents the preset value, while the dotted line denotes the solved value. Further comparison is given in Figure 16, which shows the end-effector movement trajectories in the inertial frame. One is the preset straight trajectory (denoted by the solid line) obtained using the preset data. The other one is the estimated trajectory (represented by dotted line), generated via the solved data. According to Figure 15 and Figure 16, the curve corresponding to the solved values almost coincides with that of the preset values.

**Figure 17:**
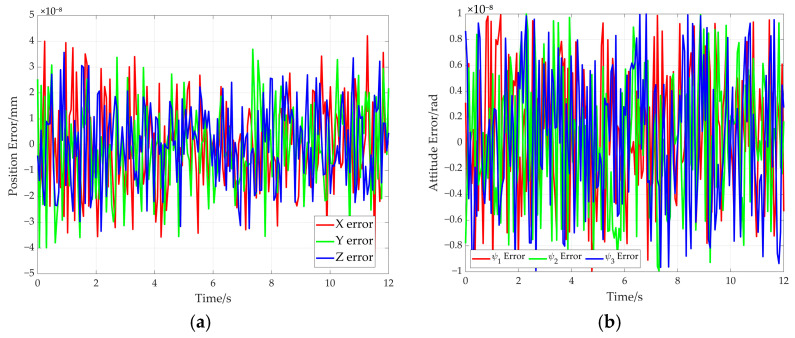
Pose errors of end-effector for the straight trajectory. (**a**) Position error; (**b**) attitude error.

**Figure 18:**
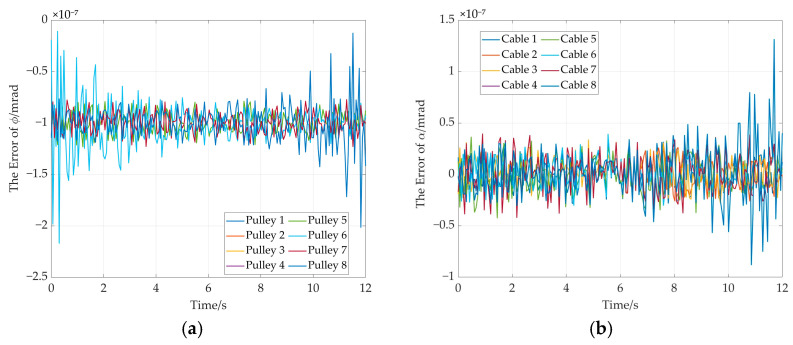
The rotation angle error and wrap angle error in the straight trajectory. (**a**) Rotation angle errors of adaptive pulleys; (**b**) cable wrap angle errors around adaptive pulleys.

The error curves of end-effector pose are shown in Figure 17. It can be seen from Figure 17a that the maximum position error remains below 4 × 10^−8^ mm. The maximum attitude error is less than 1 × 10^−8^ radians (see Figure 17b). Moreover, the rotation angle error of adaptive pulley and the wrap angle error of the cable around adaptive pulley are given in Figure 18. The maximum errors for the two parameters do not exceed 1.5 × 10^−7^ milliradians (see Figure 18a) and 2 × 10^−7^ milliradians (see Figure 18b). These simulation results demonstrate the high solution accuracy of the proposed method, thereby validating its correctness.

**Figure 19:**
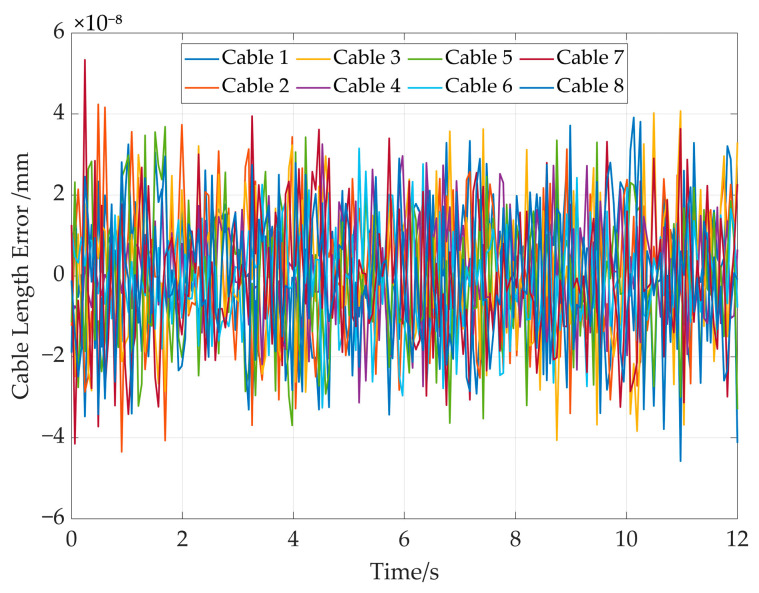
The cable length errors in the straight trajectory simulation.

The cable length errors in the simulation are shown in Figure 19; it can be seen that that the error between $l_{d}$ and l is below 6 × 10^−8^ mm. It demonstrates the high precision and effectiveness of the proposed solution approach.

#### 5.2.2. Forward Kinematics Simulation Based on the Elliptical Path via the HLMGA Algorithm

**Figure 20:**
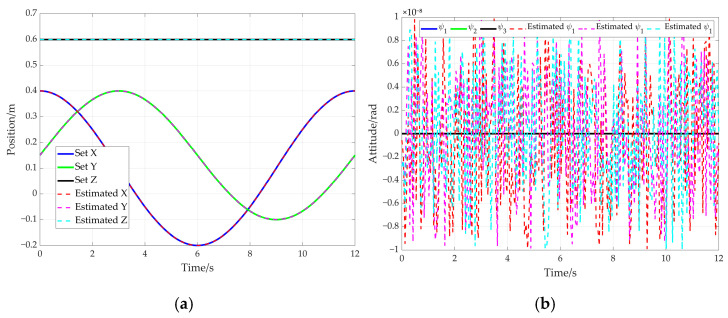
The comparison between preset values and solved values for the elliptical path. (**a**) End-effector position comparison; (**b**) end-effector attitude comparison.

**Figure 21:**
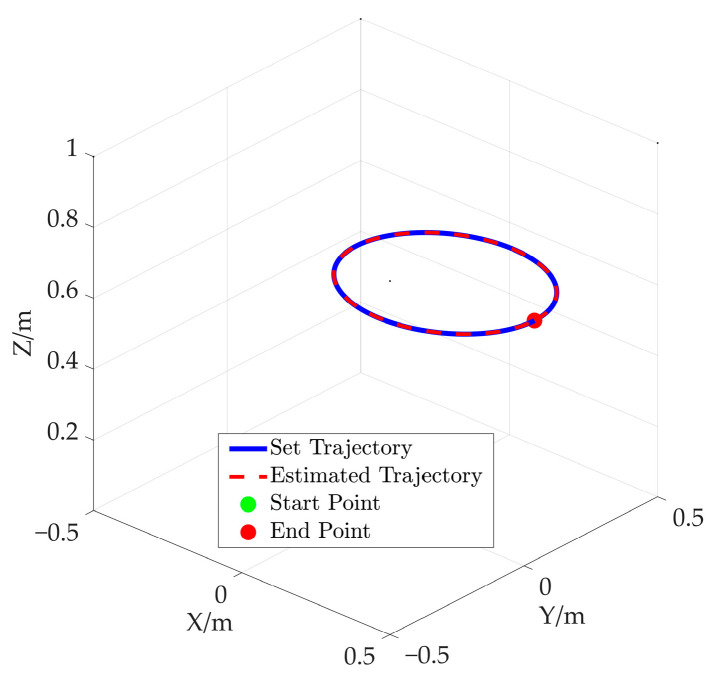
The comparison between the preset elliptical path and estimated elliptical path.

In order to further verify the proposed method, an additional simulation based on an elliptical trajectory is conducted. In this simulation, the cable length data corresponding to the ellipse are utilized to compute the end-effector’s pose. The comparison between the preset path of the end-effector and the solved path is given in Figure 20. Figure 20a represents the end-effector position comparison and Figure 20b shows the end-effector attitude comparison, where the solid line and the dotted line are preset values and solved values for the elliptical path, respectively. Furthermore, the preset trajectory and the solved trajectory are visualized in three-dimensional space, as shown in Figure 21. Based on Figure 20 and Figure 21, the curve corresponding to the solved values almost coincides with that of the preset values.

**Figure 22:**
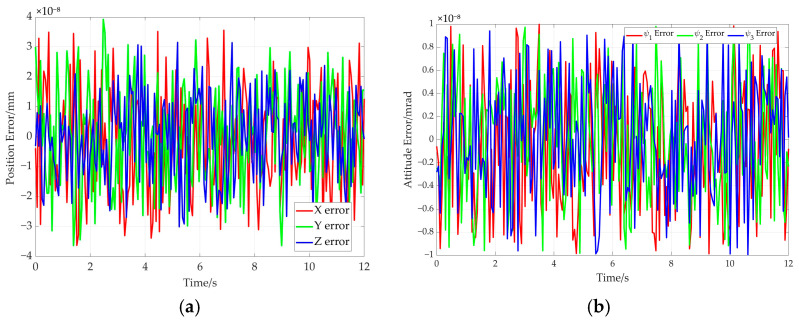
The pose errors of the end-effector for the elliptical path. (**a**) Position error; (**b**) attitude error.

**Figure 23:**
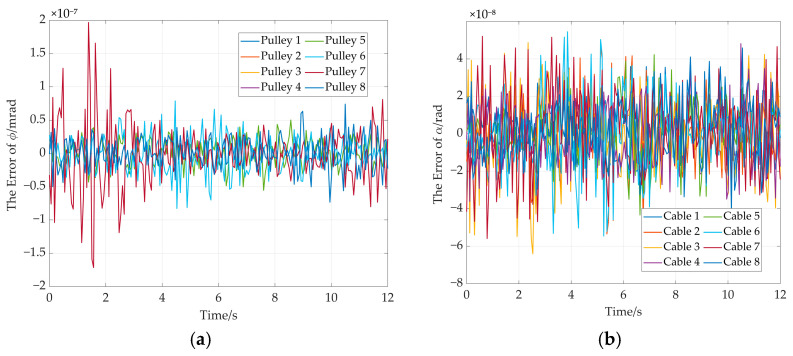
The rotation angle error and wrap angle error. (**a**) Rotation angle error of each adaptive pulley; (**b**) cable wrap angle error around the adaptive pulley.

The pose error curves of the end-effector are given in Figure 22. It can be concluded that the maximum position error is less than 5 × 10^−8^ mm (see Figure 22a). The maximum attitude error is less than 1 × 10^−8^ radians (see Figure 22b). In addition, the rotation angle error of the adaptive pulley and wrap angle error of the cable around the adaptive pulley are given in Figure 23. The maximum error of rotation angle remains below 8 × 10^−8^ milliradians and it is 2 × 10^−7^ milliradians for the cable wrap angle error. They are both less than 2.5 × 10^−7^ milliradians. These simulation results further demonstrate the high solution accuracy of the proposed method, thereby validating its effectiveness.

**Figure 24:**
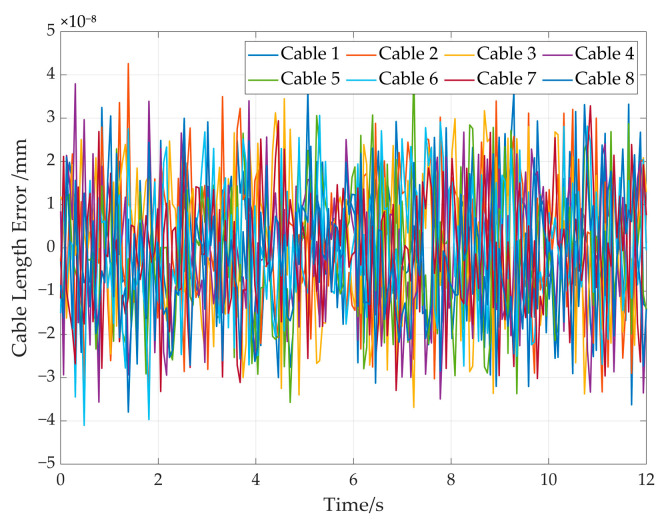
The cable length errors in the elliptic trajectory simulation.

The cable length errors in the simulation are shown in Figure 24, which shows that the error between $l_{d}$ and l is below 5 × 10^−8^ mm. It further demonstrates the high precision and effectiveness of the proposed solution approach.

#### 5.2.3. Forward Kinematics Simulation Based on the Straight Path via the PSO Algorithm

**Figure 25:**
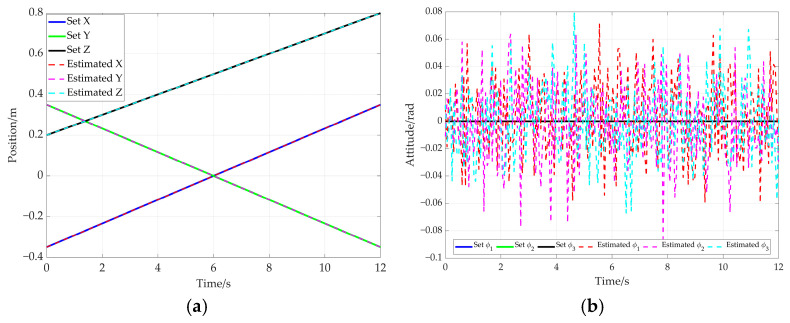
The comparison between preset values and solved values for the straight path. (**a**) End-effector position comparison; (**b**) end-effector attitude comparison.

Based on the Particle Swarm Optimization (PSO) algorithm, the comparison results between the set pose quantities and the solution values obtained using the PSO method are presented. Figure 25a shows the comparison of the end-effector position, and Figure 25b shows the comparison of the end-effector attitude. In these figures, the solid lines and dashed lines represent the preset values and the solution values of the straight path, respectively.

**Figure 26:**
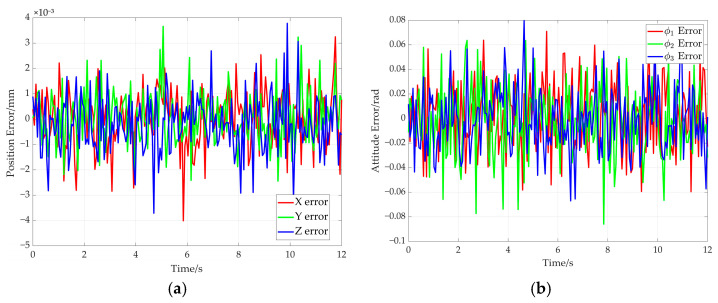
The pose errors of the end-effector for the straight path. (**a**) Position error; (**b**) attitude error.

**Figure 27:**
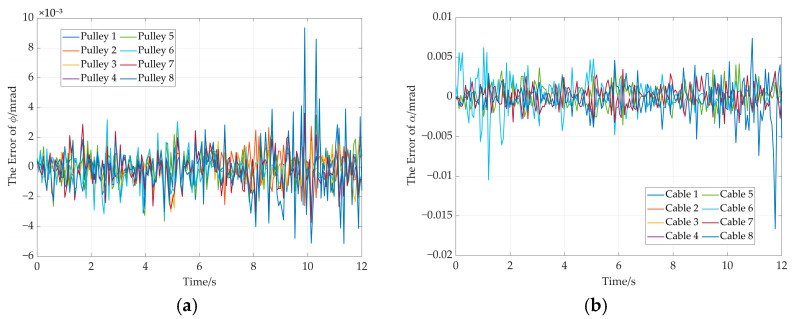
The rotation angle error and wrap angle error. (**a**) Rotation angle error of each adaptive pulley; (**b**) cable wrap angle error around the adaptive pulley.

The pose error curve of the end effector is shown in Figure 26. It can be concluded that the maximum position error is less than 4 × 10^−3^ mm (as shown in Figure 26a). The maximum attitude error is less than 6 × 10^−2^ radians (as shown in Figure 26b). Additionally, the rotation angle error of the adaptive pulley and the cable wrap angle error around the adaptive pulley are as shown in Figure 27. The maximum error in the angles of both is kept below 1 × 10^−2^ milliradians.

### 5.3. Simulation Results Analysis

To evaluate the performance of the proposed method, the results of the two aforementioned algorithms were compared, and linear and elliptical trajectories were simulated. Based on the simulation results, the following conclusions can be drawn:

(a) By performing inverse kinematics simulations on the generalized kinematic model and the improved kinematic model considering the adaptive pulley effect, we concluded that the general method would result in significant errors in the calculated cable values (more than 0.02 m). This means that the above calculated values cannot be used as cable length control data in the actual operation of the CDPR. Compared with the generalized kinematic model, the improved method can effectively solve this problem.

(b) Although the forward kinematics problem is more challenging than the inverse kinematics problem, the proposed method can also effectively handle this issue. According to the simulation results, for both linear and elliptical paths, the position error of the end effector is below 5 × 10^−8^ m. For the attitude error of the end effector, it is still less than 1 × 10^−8^ radians. However, when using the Particle Swarm Optimization algorithm to solve the forward kinematics of the linear trajectory, the result is much lower in accuracy than the HLMGA method. The simulation results demonstrate the high accuracy of these solutions, thereby verifying the overall effectiveness of the proposed method.

## 6. Conclusions

To improve the kinematic accuracy of the cable-driven parallel robot (CDPRs), a kinematic modeling and solution method based on the hybrid Levenberg–Marquardt algorithm and genetic algorithm is proposed. The structural design is analyzed to clarify the working principle of the CDPR. A generalized kinematic model and an improved kinematic model considering adaptive pulleys are established. Then, a kinematic solution method based on the hybrid Levenberg–Marquardt algorithm and genetic algorithm is developed to achieve high-precision solutions. Finally, to evaluate the effectiveness of the proposed method, a simulation system including a forward kinematic simulation model and an inverse kinematic simulation model is constructed using Matlab2020 software. It should be noted that the effectiveness of the method is fundamentally rooted in its rigorous mathematical derivation and algorithmic design, rather than in any particular software. Here, Matlab2020 is employed merely as an efficient and general-purpose numerical computing and simulation tool to execute the algorithm, solve the equations, and visualize the results. Straight line paths and elliptical paths are introduced as the expected trajectories of the end effector in the two simulation models. To demonstrate the performance of the HLMGA method, the particle swarm optimization algorithm was used to solve the forward kinematic solution of the linear trajectory for comparison. The simulation results show that, for the generalized kinematics, the error of the inverse kinematic solution is greater than 0.02 m. This indicates that the generalized kinematic model is not suitable for the CDPR with adaptive pulleys. Moreover, based on the hybrid Levenberg–Marquardt algorithm and genetic algorithm, the position and posture accuracy of the end effector are, respectively, lower than 5 × 10^−8^ mm and 1 × 10^−7^ radians. By comparing these results with the results of the particle swarm optimization algorithm, it is shown that the accuracy of the HLMGA method is relatively high. The simulation results demonstrate the high accuracy and overall effectiveness of the proposed method.

The current research only focuses on the kinematic modeling and solution of the CDPR. The dynamic characteristics and control are not investigated for the CDPR. Moreover, the effects of the elastic and nonlinear cable deformation on the kinematic accuracy are not considered in this study. Future research will address this area.

## Figures and Tables

**Table 1:** The parameters of the cable-driven parallel robot.

Item	Value	Unit
The size of the frame	1000 × 1000 × 1000	mm
The size of the end-effector	100 × 100 × 100	mm
The radius of the adaptive pulley	0.015	m
The number of driven cables	8	/
Degrees of freedom of the end-effector	6	DOF

## Data Availability

Data are contained within the article. Further inquiries can be directed to the corresponding authors.
